# Associations of Muscle Mass, Strength, and Power with Falls Among Active Community-Dwelling Older Adults

**DOI:** 10.3390/diagnostics16020283

**Published:** 2026-01-16

**Authors:** Priscila Marconcin, Joana Serpa, José Mira, Ana Lúcia Silva, Estela São Martinho, Vânia Loureiro, Margarida Gomes, Petronela Hăisan, Nuno Casanova, Vanessa Santos

**Affiliations:** 1 Insight: Piaget Research Center for Ecological Human Development, Instituto Piaget, 2805-059 Almada, Portugal; priscila.marconcin@ipiaget.pt (P.M.); joana.serpa@ipiaget.pt (J.S.); ana.lucia@ipiaget.pt (A.L.S.); nuno.martins@ipiaget.pt (N.C.); vanessa.santos@ipiaget.pt (V.S.); 2 Faculty of Health Sciences, Universidad Autónoma de Chile, Providencia 7500912, Chile; 3 Egas Moniz Center for Interdisciplinary Research (CiiEM), Egas Moniz School of Health & Science, Caparica, 2829-511 Almada, Portugal; jmira@egasmoniz.edu.pt; 4 Higher Institute of Intercultural and Transdisciplinary Studies (ISEIT), Instituto Piaget, 2805-059 Almada, Portugal; estelabsmfisio@gmail.com; 5 Department of Arts, Humanities and Sports, School of Education, Polytechnic University of Beja, 7800-295 Beja, Portugal; margarida.gomes@ipbeja.pt; 6 Sport Physical Activity and Health Research & Innovation Center (SPRINT), 7800-295 Beja, Portugal; 7 Department of Physical Education and Sport, “1 Decembrie 1918” University of Alba Iulia, 510009 Alba Iulia, Romania; petronela.haisan@uab.ro; 8 Interdisciplinary Center for the Study of Human Performance (CIPER), Faculty of Human Kinetics, University of Lisbon, 1495-751 Lisboa, Portugal

**Keywords:** falls, ageing, muscle strength, muscle power, functional performance

## Abstract

**Background/Objectives**: Falls are a leading cause of morbidity and mortality in older adults, even among those who are physically active. This study examined the associations between skeletal muscle mass, muscle strength, and muscle power and fall risk in physically active, community-dwelling older adults. **Methods**: A cross-sectional analysis was conducted with 280 participants (71.9 ± 5.3 years; 75% women) enrolled in the Stay Up–Falls Prevention Project. Assessments included skeletal muscle mass (anthropometric prediction equation), handgrip strength, lower limb strength and power (Five Times Sit-to-Stand test, 5×STS), and fall history over the past 12 months. Muscle power was calculated from 5×STS performance using the equation proposed by Alcazar and colleagues. Logistic regression models and receiver operating characteristic (ROC) curve analyses were performed. **Results**: Overall, 26.4% of participants reported at least one fall in the previous year, with a higher prevalence among women (28.9%) than men (18.8%). Fallers showed significantly lower handgrip strength (23.1 vs. 25.4 kg, *p* = 0.022) and poorer lower limb strength (9.2 vs. 8.7 s, *p* = 0.007) compared with non-fallers. However, no significant differences were found for skeletal muscle mass or sit-to-stand–derived power. In multivariable models adjusted for age, sex, body mass index, comorbidities, and medications, lower limb strength remained the only independent variable associated with fall status (OR = 1.78, 95% CI: 1.11–2.85, *p* = 0.016). ROC analysis confirmed fair discriminative capacity for 5×STS performance (AUC = 0.616, *p* = 0.003), with an optimal cut-off of 8.62 s (sensitivity = 78.4%, specificity = 33.0%). Handgrip strength, muscle mass, and power did not show independent associations with fall status. **Conclusions**: These findings indicate that the 5×STS test provides a simple, cost-effective, and functional indicator for fall-risk stratification in physically active older adults. Clinicians should consider the 5×STS as a sensitive functional indicator that contributes to fall risk stratification, ideally combined with complementary assessments (e.g., balance, gait, cognition) to improve risk stratification and guide preventive interventions in ageing populations.

## 1. Introduction

Aging is a complex biological process characterized by progressive and multidimensional changes in physiological systems, which contribute to increased vulnerability, functional decline, and a higher risk of disability and mortality among older adults [1]. Among the various biological systems affected by aging, the neuromuscular system undergoes some of the most critical and early changes, directly impacting mobility, autonomy, and quality of life [2]. Such neuromuscular impairments, particularly reductions in skeletal muscle mass (SMM), strength, and power, have been consistently associated with impaired balance and an increased fall risk in older populations [3,4].

The prevalence of falls among older adults is estimated at 26.5% worldwide and 23.4% in Europe [5]. Between 2010 and 2018, in Portugal, 65.4% of hospital admissions were attributable to falls with the majority occurring among individuals aged 65 years and older [6]. Importantly, falls impose a substantial economic burden on healthcare systems, considering their strong association with morbidity, disability, hospitalization, institutionalization, and mortality [7]. Moreover, even among older adults with relatively high levels of physical function, falls can compromise autonomy, diminish confidence, and negatively impact quality of life [8].

Falls in older adults result from the interaction of multiple risk factors, which are commonly classified as intrinsic (biological) or extrinsic (environmental) [9]. Intrinsic determinants includes age, sex, and age-related physiological alterations, as visual and auditory impairments, balance disorders, increased fat mass, decreased SMM, and other chronic conditions (e.g., cardiovascular diseases). Within this context, neuromuscular impairments, particularly declines in SMM, strength, and power are considered key determinants of mobility decline and the increased fall risk [3,4]. Additionally, the presence of multiple comorbidities and polypharmacy further increases the likelihood of falls, both through direct physiological effects and interactions with declining neuromuscular function [10,11].

Historically, the loss of SMM in association with impaired physical function or muscle strength, classified as sarcopenia, has been considered the primary risk factor for age-related declines in physical and autonomy [12]. However, evidence suggests that reduced muscle strength (dynapenia) and potentially, muscle power, may contribute to more frequent and at faster rates to physical and autonomy decline than SMM, and strongly associated with the onset of functional limitations and adverse outcomes [13,14].

Muscle power, defined as the product of force and contraction velocity, has emerged as a stronger predictor of physical performance in older adults than maximal strength alone [15,16]. Overall, muscle power is especially relevant in daily tasks that require rapid force generation, such as rising from a chair, preventing a fall, or crossing a street, thus making its decline more functionally impactful than strength deficits itself [2,13], reductions in both muscle strength and power are not only markers of functional decline but also recognized as critical risk factors for falls, highlighting the importance of their assessment to study falls prevention [3,4]. Evidence shows that muscle power declines earlier and more steeply with age than strength or SMM and has stronger associations with mobility limitations and fall risk [13,14,15]. On the other hand, lower muscle strength associates moderately with increased fall risk in community-dwelling older adults [17]. Moreover, older adults who had fallen in the previous year were shown to have significantly lower muscle mass [18,19].

However, emerging evidence suggests that muscle weakness in older individuals is primarily attributed to a decline in muscle quality rather than the loss of muscle mass itself. Muscle quality refers to the ability of muscle fibers to generate force efficiently and plays a pivotal role in the age-related decline in strength and power, independent of SMM [20]. Additionally, age-related changes in muscle fiber type and contractile properties undoubtedly contribute to neuromuscular deterioration [21].

SMM, strength, and particularly power is essential to maintaining function and therefore independence in older adults [22,23]. Regular physical activity is widely recognized as a protective factor against functional decline and falls [24,25]. Nevertheless, evidence shows that falls remain prevalent even among physically active and independent older adults [26]. This suggests that neuromuscular impairments may persist despite engagement in regular exercise, highlighting the need to investigate specific predictors of fall risk in this population. Understanding why active older adults still fall is essential to develop targeted interventions beyond general physical activity.

Therefore, this study aimed to examine the associations of SMM, strength, and muscle power with the fall risk in physically active older adults. Specifically, we sought to: (a) compare neuromuscular characteristics between fallers and non-fallers, (b) identify variables independently associated with falls through multivariate models, and (c) evaluate the discriminative ability of muscle-related variables using receiver operating characteristic (ROC) curve analysis. By addressing these objectives, the present study contributes to clarify the role of neuromuscular function in the fall risk and provides evidence to inform fall prevention strategies for physically active community-dwelling older adults. From a clinical perspective, identifying objective variables and cut-off points for neuromuscular measures (muscle strength and power), is crucial for risk stratification and fall-prevention guidelines in older adults [27,28].

## 2. Materials and Methods

This is a cross-sectional study integrated in the cohort Stay Up–Falls Prevention Project, an ongoing initiative launched in 2021 that investigates the determinants of fall risk among physically active older adults. The project encompasses community-based assessments and interventions, developed in collaboration with local municipal health programs. Its overarching aim is to contribute to the development of public health strategies focused on fall prevention and the promotion of healthy ageing [29]. The study protocol received ethical clearance from the Ethics Committee of the Piaget Institute (reference: P02-S40-11 January 2023). Prior to participation, all individuals provided written informed consent in accordance with ethical guidelines. The research was conducted in strict compliance with internationally recognized ethical principles, including the Declaration of Helsinki, the Belmont Report, and the Ethical Standards for Research in Sport and Exercise Science. Data confidentiality and participant anonymity were rigorously maintained throughout the study.

### 2.1. Participants

All participants were recruited from a community-based exercise program implemented by a municipal authority in the Lisbon metropolitan area (Portugal), consisting of two weakly sessions of multicomponent physical. The multicomponent exercise program included a combination of balance, strength, and aerobic exercises, in accordance with current physical activity recommendations for older adults. All individuals enrolled in the program were informed about the study one week before the assessment period and were invited to voluntarily participated. No a priori sample size was performed; instead, all eligible individuals who agreed to participate were included in the study. Participants were instructed to maintain their usual regular physical activity routines, dietary habits, and hydration levels during the two days preceding data collection. Assessments were conducted in the morning and included demographic, anthropometric and performance tests. Participants were included in the study if they: (i) were aged 65 years or older; (ii) were not participating in any other physical exercise program; (iii) are able to move independently without assistance; (iv) had the ability to understand the study’s instructions and protocols; and (v) provided written informed consent. Conversely, older adults were excluded if they had contraindications for physical exercise or are unable to complete the physical assessment protocol.

### 2.2. Instruments and Variables

Demographic and anthropometric data was obtained, including sex, age, number of comorbidities and current medication use, weight and height. Body weight was measured using a calibrated mechanical scale (SECA 761; Bacelar & Irmão Lda, Porto, Portugal) and height was assessed with a stadiometer (Seca GmbH & Co. KG; dimensions: 337 mm × 2165 mm × 590 mm; Hamburg, Germany). Body mass index (BMI) was calculated as body weight (kg) divided by height squared (m^2^).

#### 2.2.1. Assessment of Falls

Fall history was evaluated based on the recommendations of the American Geriatrics Society and the British Geriatrics Society [30], participants were asked: (1) ‘How many times have you fallen in the past 12 months?’ If the response was affirmative, follow-up questions included: (2) ‘Did you require medical attention as a result of the fall?’ and (3) ‘Did the fall cause any difficulties with walking or balance?’ Participants could report multiple events, and the total number of falls was documented accordingly. For analytical purposes, participants reporting one or more falls in the previous 12 months were classified as fallers. Recurrent fallers were not analyzed separately due to sample size considerations and to maintain sufficient statistical power for multivariable analyses.

#### 2.2.2. Skeletal Muscle Mass

SMM was estimated using the anthropometric prediction equation proposed by Lee et al. [31], which was developed and cross-validated against whole-body magnetic resonance imaging. The model incorporates body weight (kg), height (m), age (years), sex (male = 1; female = 0), and race (white/hispanic = 0; african american = +1.9; Asian = −1.6). The equation is expressed as:

SMM (kg) = 0.244 × weight + 7.8 × height + 6.6 × sex − 0.098 × age + race − 3.3

#### 2.2.3. Handgrip Strength

Handgrip strength (HGS) was assessed using a mechanical hand dynamometer (Saehan SH5001; Saehan Corporation, Changwonsi, Republic of Korea), a widely recognized spring-type device for the evaluation of manual strength. Prior to data collection, the device was checked to confirm mechanical accuracy and consistant resistance.

Measurements were performed on the dominant hand, with the dynamometer individually adjusted to the individual’s hand size. Participants were seated during testing, maintaining the shoulder in a neutral position, the arm aligned with the torso, the forearm extended, and the wrist in a neutral position. Three maximal voluntary contractions were performed, and the highest value was recorded. Each contraction lasted approximately three seconds per attempt, with sixty seconds of rest interval were provided between the three trials [32].

#### 2.2.4. Lower Limb Strength and Power

The Five Times Sit-to-Stand Test (5×STS), a component of the Short Physical Performance Battery (SPPB), as originally described by Guralnik et al. [33], was utilized to assess lower limb strength (LLS) and power. Participants began the test seated on a standard-height chair, with arms crossed over the chest and lower limbs positioned approximately at a 90° angle at the hip, knee, and ankle joints. Feet were placed parallel and flat on the floor. The task consisted of standing up to full knee extension and returning to the seated position, repeating this cycle five times consecutively, as quickly as possible, without using the upper limbs. To evaluate LLS was used a stopwatch, and the total duration to complete the 5 repetitions was recorded. Power was based on 5×STS and calculated based on the equation proposed by Alcazar et al. [16]:

$${\bar{P}}_{5\times STS}=\frac{0.9\;\cdot \;m\;\cdot g\;\cdot [h_{p}\;\cdot 0.5\;-h_{c}]}{t_{5\times STS}}$$

where $\bar{P}$_(5×_*_STS_*_)_ is the average power over 5 repetitions, *m* the body mass of the individual, *g* the gravitational acceleration, $h_{p}$ the height of the participant, $h_{c}$ the height of the chair (i.e., 43 cm), $t_{5\times STS}\;$ represents the average time per repetition, calculated as the total time to complete the five sit-to-stand repetitions divided by five.

### 2.3. Statistical Analysis

Descriptive statistics are presented as mean ± standard deviation (SD) for continuous variables and frequencies and percentages for categorical variables. Independent t-tests were used to compare differences between sexes and between fallers and non-fallers. Effect sizes for t-test were calculated using partial eta-squared (η^2^; *p*) to estimate the strength of relationships between variables and the results were considered as “small”, “medium” or “large” effect sizes if 0.01 ≤ η^2^; *p* < 0.06, 0.06 ≤ η^2^; *p* < 0.14 and η^2^; *p* ≥ 0.14, respectively [34]. Logistic regression models were applied to examine the associations between neuromuscular-related variables (SMM, HGS, lower limb strength, and power) and fall risk, first in crude models, followed by models adjusted for age and sex, and finally in multivariate models adjusted for all covariates. Multicollinearity among neuromuscular variables was examined using variance inflation factors (VIF) and pairwise correlation coefficients. All VIF values were below 5.0, suggesting acceptable levels of collinearity. The discriminative ability of significant variables was further evaluated using ROC curve analysis, with the area under the curve (AUC) and 95% confidence intervals (CI). The optimal cutoff value for strength assessed with the five-repetition sit-to-stand test was determined using the Youden Index, which maximizes the sum of sensitivity and specificity of the ROC curve [35]. This approach identifies the threshold that provides the best balance between correctly identifying true positives and minimizing false positives. Sensitivity and specificity values corresponding to this cutoff were reported. Statistical significance was set at *p* < 0.05.

## 3. Results

A total of 280 participants were included in the analysis (*n* = 211 female and *n* = 69 male; mean age = 71.88 ± 5.35 years). Men showed significantly higher values for body weight, height, SMM, HGS and power compared to women (*p* < 0.001). No statistically significant differences were observed between sexes for age, BMI, number of medications, number of comorbidities, and lower limb strength (Table 1).

Table 2 presents the characteristics related to fall risk. Among the 280 participants, 26.4% (*n* = 74) reported at least one fall in the past 12 months, with a higher proportion observed among females (28.9%) compared to males (18.8%).

Table 3 shows the comparison between fallers and non-fallers in physical variables. Fallers demonstrated significantly lower HGS compared with non-fallers (23.15 ± 6.49 vs. 25.39 ± 7.34 kgf, *p* = 0.022, d = 0.313), representing a small-to-moderate effect size. In addition, fallers performed significantly worse in the LLS test, with higher time to complete the test than non-fallers (9.24 ± 1.72 vs. 8.65 ± 1.53 s, *p* = 0.007, d = −0.371, moderate effect size). No significant differences were observed between groups for SMM (20.53 ± 5.05 vs. 19.89 ± 4.98 kg, *p* = 0.353, d = 0.126) or power (261.98 ± 71.89 vs. 248.88 ± 87.42, *p* = 0.206, d = 0.172).

Table 4 presents the results of logistic regression models examining associations between neuromuscular variables and fall status. In crude analyses, lower HGS and longer time in 5×STS were associated with higher odds of falling. After adjustment for age and sex, only LLS remained significant (OR = 1.21; 95% CI: 1.03–1.43, *p* = 0.019). In the fully adjusted multivariate model, LLS remained independently associated with fall status (OR = 1.78; 95% CI: 1.11–2.85, *p* = 0.016), whereas SMM, HGS, and power were not significantly associated.

The ROC curve analysis showed that only LLS and HGS presented significant discriminative ability for fall status. LLS showed fair but limited discriminative accuracy, with an AUC of 0.616 (95% CI: 0.539–0.693, *p* = 0.003), while HGS presented poor discriminative performance (AUC = 0.397, 95% CI: 0.320–0.474, *p* = 0.009). SMM (AUC = 0.470, 95% CI: 0.393–0.547, *p* = 0.393) and power (AUC = 0.424, 95% CI: 0.346–0.503, *p* = 0.054) did not show significant ability to differentiate fallers from non-fallers (Table 5).

**Figure 1:**
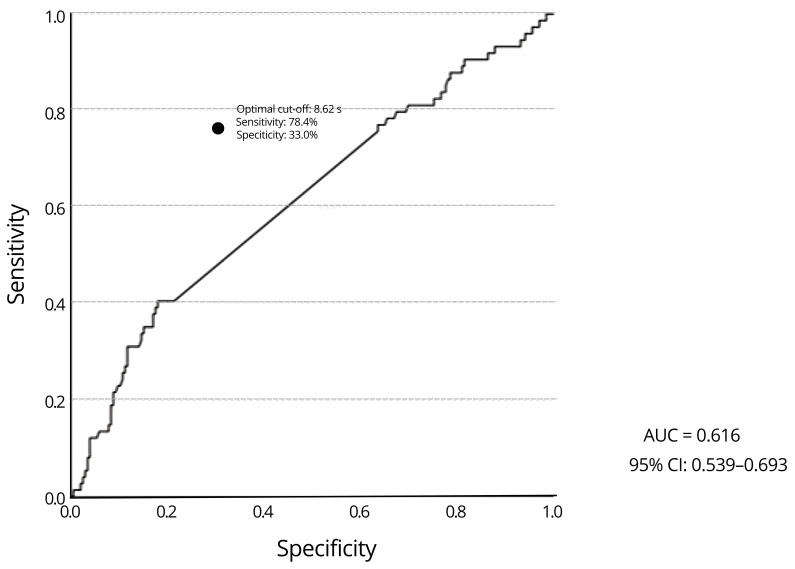
ROC curve for sit-to-stand time associated with fall risk.

Figure 1 represents the ROC curve analysis, which revealed that was LLS significantly associated with fall risk in physically active older adults. The (AUC) was 0.616 (95% CI: 0.539–0.693, *p* = 0.003), indicating a modest but significant discriminatory ability to differentiate fallers from non-fallers. Although sensitivity was high, the low specificity substantially limits its clinical applicability when used in isolation. The AUC value above 0.60 suggests that LLS has a fair discriminative capacity, although it does not reach the threshold generally considered strong (AUC ≥ 0.70). The optimal cutoff point for the 5×STS was 8.62 s, with 78.4% sensitivity and 33.0% specificity, indicating that longer completion times are associated with increased fall risk.

## 4. Discussion

In a community-dwelling cohort of physically active older adults (*n* = 280), 26.4% reported a fall over 12 months. LLS, measured by the 5×STS test, was the only independent neuromuscular variable associated with fall risk, whereas SMM, HGS, and muscle power were not significant after multivariable adjustment. HGS was lower in fallers, but its association attenuated after accounting for age, sex, BMI, comorbidities, medications, and other muscle-related variables. ROC curve analysis for 5×STS indicated fair but limited discriminative accuracy (AUC = 0.616) with an optimal cut-off of 8.62 s, showing high sensitivity but low specificity.

This study holds relevance as it examined a cohort of physically active older adults, a group often assumed to be at reduced risk, but who nonetheless remain susceptible to falls. The relatively high and homogeneous level of physical activity in this sample may have attenuated inter-individual variability in neuromuscular performance, potentially limiting the ability to detect stronger associations between muscle-related variables and fall status. In physically active populations, physical function may cluster within a narrower range, reducing contrast between fallers and non-fallers compared with more heterogeneous or sedentary cohorts. While regular physical activity is consistently recognized as a protective factor against functional decline and falls, it does not fully mitigate the intrinsic vulnerabilities associated with ageing, such as neuromuscular impairments, multimorbidity, and polypharmacy [9,25]. In our sample, the prevalence of falls over the past 12 months was 26.4%, closely aligning with the global prevalence of 26.5% and slightly exceeding the European average of 23.4% [5]. As well as prior Portuguese data reporting of 20–23% annual fall incidence, however, only 7% of fallers required medical intervention, while the majority of events were managed without healthcare assistance [6]. This finding indicates that although falls are relatively common in physically active older adults, their severity may be attenuated compared to more vulnerable populations, such as frail or institutionalized individuals, in whom falls frequently lead to hospitalization, loss of independence, and increased mortality [7,36].

Our findings highlight the growing consensus that measures of SMM quantity are insufficient to capture fall risk, which may include other parameters such as muscle power, thereby providing a more comprehensive assessment of mobility relevant to real-life fall scenarios [16,37]. The discriminative ability of 5×STS for LLS, in this study aligns with prior evidence demonstrating its sensitivity, but not necessarily high specificity, in identifying older adults at heightened risk of disability, institutionalization, and falls [1,33]. By contrast, HGS, although is lower in fallers, lost significance once covariates were considered in the associations of fall models, suggesting it may be a less specific indicator of fall risk compared to LLS, particularly in physically active populations. Taken together, these results highlight that dynamic, task-specific functional assessments may outperform isolated muscle measures in discriminating fall risk among active community-dwelling older adults.

Although muscle power was not significant associated with falls in the present analyses, its assessment remains conceptually relevant. It should be acknowledged that both LLS and sit-to-stand-derived muscle power originate from the same functional test (5×STS), which introduces conceptual and methodological overlap. This shared measurement basis may result in collinearity and partial construct redundancy, potentially influencing the results of multivariable models. Although muscle power is theoretically more sensitive to age-related functional decline, it did not emerge as an independent variable in the multivariable models. This may be partly explained by the strong correlation between 5×STS-derived power and LLS, as well as by the indirect estimation of power through predictive equations rather than direct biomechanical assessment. Muscle power declines earlier and more rapidly than muscle strength with aging and has been identified as a critical determinant of mobility and independence [15,16]. More recently, the concept of powerpenia has been proposed to denote the age-related loss of muscular power as a distinct biomarker of functional decline and fall risk [2]. In this context, the ability to estimate power from the 5×STS, in parallel with LLS, offers a pragmatic approach to capturing two complementary aspects of neuromuscular performance within a single functional test. Although the present study does not direct evaluated muscle power decline, future longitudinal studies incorporating direct biomechanical assessments of muscle power are needed to clarify the role of power decline and provide earlier indications of vulnerability to functional limitations and falls.

In our study, the cut-off value of 8.62 s on the 5×STS was substantially lower than those reported in broader or more heterogeneous cohorts, highlighting both the superior baseline capacity of physically active older adults and the need for tailored thresholds to improve the accuracy of fall risk screening in this group. For example, Buatois et al. [38] identified a threshold of 15 s as indicator for risk stratification of recurrent falls in community-dwelling older people, while Albalwi and Alharbi [39] reported cut-offs of >11.5 s for individuals aged 65–74 years and >12.10 s for those aged 75 and above. Similarly, Ramírez-Vélez et al. [40] provided reference values for 5×STS stratified by age and sex, further highlighting that time-based thresholds tend to be higher in less active or older populations. Taken together, these comparisons suggest that the markedly lower cut-off observed in our cohort reflects superior baseline functional capacity. However, the identified cut-off should not be interpreted as a clinically actionable threshold, but rather as a population-specific reference value. The modest discriminative accuracy of the 5×STS (AUC = 0.616), despite being statistically significant, indicates limited clinical utility when used in isolation for fall risk stratification. This reinforces the notion that the 5×STS should be regarded as a sensitive but non-specific functional screening indicator, best applied alongside complementary assessments (e.g., SMM quantity, balance, gait, or cognitive testing) rather than as a stand-alone screening tool.

Sex and age-related considerations merit explicit discussion. In our cohort, men exhibited higher upper-body strength and muscle power compared with women, yet 5×STS did not significantly differ between sex, suggesting that LLS may converge among physically active older adults. However, empirical data indicate that fall risk is differentially patterned by sex and age. For instance, Soh and Won [41] found that fall and fracture incidence were higher among older women than men, with sex-specific associations between sarcopenia components and fall outcomes. Similarly, Suh et al. [42] reported divergent fall-risk factors based on gender, nutritional status and instrumental activity daily basis living dependence in women versus medication use and stair-climbing ability in men. These findings underscore the limitation of applying uniform cut-offs across demographic groups. Tailoring 5×STS thresholds by sex and age could refine screening accuracy, enhancing both sensitivity and specificity in fall-risk identification.

From a clinical perspective, the 5×STS may be considered a practical and cost-effective functional test that contributes to fall risk stratification, particularly, in active older adults. However, its limited discriminative accuracy and very low specificity substantially restrict its use as a stand-alone screening tool in real-world settings, as this may lead to a high number of false-positive classifications. Instead of isolated measures of muscle mass or HGS, the 5×STS integrates LLS, balance, coordination, and transitional mobility, all of which are directly relevant to fall scenarios. Accordingly, the identified cut-off value should be interpreted with caution and only within the context of broader, multidimensional assessment frameworks. Moreover, these results support the design of tailored exercise interventions emphasizing not only LLS but also movement velocity and task-specific functional training, which have been shown to reduce fall incidence and improve physical performance [2,25]. Implementing these approaches may enhance preventive strategies, ultimately helping to preserve independence and quality of life in ageing populations.

Several limitations of the present study should be acknowledged. First, SMM was not directly measured using gold-standard methods such as dual-energy X-ray absorptiometry or magnetic resonance imaging. Instead, it was estimated through predictive equations based on anthropometric variables (weight, height, sex, and race), which may introduce measurement error and limit the precision of associations with fall risk. Similarly, muscle power was estimated using a predictive equation based on sit-to-stand performance, which does not directly capture contraction velocity or force production. Although this approach has been previously validated, it may not fully capture the complexity of neuromuscular power as measured through biomechanical or laboratory-based methods. Furthermore, the cross-sectional design and the sampling type and dimension exclude any inference of causality. Additionally, reverse causality cannot be excluded, as previous falls may have contributed to poorer neuromuscular performance at the time of assessment. Therefore, the results should be interpreted as associations only, and not as predictive or causal determinants of falls. The sample was recruited through convenience procedures and consisted exclusively of active community-dwelling older adults. Consequently, the finding may not be generalizable to sedentary, frail, or institutionalized populations, who typically present greater neuromuscular impairment and higher fall risk. In addition, fall events were assessed retrospectively through self-report over a 12-month period, which is subject to recall bias and may underestimate minor or forgotten incidents, particularly among physically active older adults. This may have led to misclassification between fallers and non-fallers, potentially attenuating observed associations. Moreover, falls were treated as a dichotomous and homogeneous outcome, without accounting for differences in frequency or severity, which may reduce sensitivity to detect functional and neuromuscular differences. Finally, the study did not account for other relevant fall-related risk factors such as cognitive status, sensory impairments, environmental hazards, or fear of falling, which may act as important confounders. Taken together, these methodological and sampling considerations should be considered when interpreting the present results.

## 5. Conclusions

This study shows that in physically active community-dwelling older adults, LLS derived from the 5×STS test was the only independent neuromuscular variable associated with fall risk. In contrast, SMM, HGS, and muscle power were not significant after adjustment for covariates. The derived cut-off of 8.62 s reflects better functional capacity compared with less active cohorts. However, its modest discriminative accuracy (AUC = 0.616) indicates that the test should not be used alone for risk stratification. Furthermore, the cross-sectional design excludes any inference of causality, restricting interpretation to associations rather than predictive or causal relationships. Instead, the 5×STS can be used as a sensitive first-line indicator of fall risk, complemented by other assessments targeting balance, cognition, and comorbidity. Future studies should validate sex- and age-specific thresholds in the assessment of fall risk and further investigate the role of muscular power decline in the fall trajectories. Our findings support the use of the 5×STS as a functional indicator in primary care and community settings, though it should be complemented by other assessments to ensure accurate fall risk stratification in physically active older adults.

## Figures and Tables

**Table 1:** Sample characteristics, total and stratified by sex.

Variables	Total (*n* = 280)	Male (*n* = 69)	Female (*n* = 211)	*p*-Value
Age (years)	71.88 ± 5.35	73.07 ± 6.10	71.49 ± 5.04	0.055
Body Weight (kg)	68.56 ± 11.32	75.19 ± 10.84	66.40 ± 10.64	<0.001
Height (m)	1.58 ± 0.07	1.66 ± 0.06	1.55 ± 0.05	<0.001
BMI (kg/m^2^)	27.29 ± 4.02	27.21 ± 3.93	27.32 ± 4.06	0.840
No. Medications	3.35 ± 2.55	3.79 ± 2.61	3.20 ± 2.53	0.105
No. Comorbidities	2.45 ± 1.73	2.66 ± 2.03	2.33 ± 1.62	0.216
SMM (kg)	20.36 ± 5.03	27.47 ± 3.02	18.03 ± 2.96	<0.001
HGS (kgf)	24.80 ± 7.18	33.43 ± 7.38	21.97 ± 4.29	<0.001
LLS (s)	8.80 ± 1.60	8.54 ± 1.44	8.89 ± 1.65	0.087
Power (W)	258.51 ± 76.36	323.31 ± 83.34	237.33 ± 60.56	<0.001

Note: Data are expressed as mean ± standard deviation (SD). Abbreviations: BMI, Body Mass Index; HGS, Handgrip Strength; LLS, Lower limb strength; *n*, sample; No., Number; SMM, Skeletal Muscle Mass.

**Table 2:** Fall risk characteristics, total and stratified by sex.

Variables	Total (*n* = 280)	Male (*n* = 69)	Female (*n* = 211)
No. Falls in the last 12 months, M (±SD)	0.52 ± 1.25	0.26 ± 0.61	0.61 ± 1.38
Falls in the last 12 months, *n* (%)			
Yes	74 (26.4)	13 (18.8)	61 (28.9)
No	206 (73.6)	56 (81.2)	150 (71.1)
Require intervention, *n* (%)			
Yes	21 (7.5)	6 (8.7)	15 (7.1)
No	259 (92.5)	63 (91.3)	196 (92.9)
Have balance/gait problems, *n* (%)			
Yes	116 (41.4)	21 (30.4)	95 (45.0)
No	164 (58.6)	48 (69.6)	116 (55.0)

Abbreviations: M, mean; *n*, sample; No., number; SD, standard deviation; %, percentage.

**Table 3:** Comparison of skeletal muscle mass and neuromuscular variables between fallers and non-fallers.

Variables	Non-Fallers (*n* = 206)	Fallers (*n* = 74)	*t*	*p*-Value	Cohen’s d
SMM (kg)	20.53 ± 5.05	19.89 ± 4.98	0.93	0.353	0.126
HGS (kgf)	25.39 ± 7.34	23.15 ± 6.49	2.31	0.022	0.313
LLS (s)	8.65 ± 1.53	9.24 ± 1.72	−2.73	0.007	−0.371
Power (W)	261.98 ± 71.89	248.88 ± 87.42	1.26	0.206	0.172

Data are expressed as the mean ± standard deviation. Abbreviations: HGS, Handgrip Strength; LLS, Lower limb strength; *n*, sample; SMM, Skeletal Muscle Mass.

**Table 4:** Association between SMM and neuromuscular variables between fallers and non-fallers.

Variables	Crude OR (95% CI)	*p*-Value	Adjusted OR ^1^ (95% CI)	*p*-Value	Final Model ^2^ OR (95% CI)	*p*-Value
SMM (kg)	0.97 (0.92–1.02)	0.975	1.07 (0.97–1.18)	0.168	0.77 (0.51–1.16)	0.219
HGS (kgf)	0.95 (0.91–0.99)	0.023	0.96 (0.91–1.01)	0.161	0.94 (0.88–1.01)	0.119
LLS (s)	1.24 (1.05–1.47)	0.008	1.21 (1.03–1.43)	0.019	1.78 (1.11–2.85)	0.016
Power (W)	0.99 (0.99–1.00)	0.207	1.00 (0.99–1.00)	0.882	1.01 (0.99–1.03)	0.062

^1^ Adjusted model controlled for age and sex. ^2^ Final model included all muscle-related variables (SMM, HGS, LLS, Power) plus age, sex, BMI, number of comorbidities, and number of medications. Abbreviations: BMI, Body Mass Index; CI, confidence intervals; HGS, Handgrip Strength; LLS, Lower limb strength; OR, Odds ratios; SMM, Skeletal Muscle Mass; %, percentage.

**Table 5:** ROC analysis for variables associated with fall risk.

Variables	AUC	95% CI	*p*-Value
SMM (kg)	0.470	0.393–0.547	0.393
HGS (kgf)	0.397	0.320–0.474	0.009
LLS (s)	0.616	0.539–0.693	0.003
Power (W)	0.424	0.346–0.503	0.054

Abbreviations: AUC, Area under the curve; CIs, confidence intervals; HGS, Handgrip Strength; LLS, Lower limb strength; SMM, Skeletal Muscle Mass; %, percentage.

## Data Availability

The original contributions presented in this study are included in the article. Further inquiries can be directed to the corresponding author.

## Abbreviations

The following abbreviations are used in this manuscript:

AUC	Area under the curve
BMI	Body mass index
CIs	Confidence intervals
5×STS	Five Times Sit-to-Stand Test
HGS	Handgrip strength
ROC	Receiver operating characteristic
SPPB	Short Physical Performance Battery
SD	Standard deviation
SMM	Skeletal muscle mass
W	Sit-to-stand Power
